# Transformations of Monoterpenes with the *p*-Menthane Skeleton in the Enzymatic System of Bacteria, Fungi and Insects

**DOI:** 10.3390/molecules25204840

**Published:** 2020-10-20

**Authors:** Małgorzata Grabarczyk, Wanda Mączka, Anna K. Żołnierczyk, Katarzyna Wińska

**Affiliations:** Department of Chemistry, Wrocław University of Environmental and Life Sciences, Norwida 25, 50-375 Wrocław, Poland; anna.zolnierczyk@upwr.wroc.pl

**Keywords:** biotransformation, bacteria, fungi, yeast, insects, terpenes

## Abstract

The main objective of this article was to present the possibilities of using the enzymatic system of microorganisms and insects to transform small molecules, such as monoterpenes. The most important advantage of this type of reaction is the possibility of obtaining derivatives that are not possible to obtain with standard methods of organic synthesis or are very expensive to obtain. The interest of industrial centers focuses mainly on obtaining particles of high optical purity, which have the desired biological properties. The cost of obtaining such a compound and the elimination of toxic or undesirable chemical waste is important. Enzymatic reactions based on enzymes alone or whole microorganisms enable obtaining products with a specific structure and purity in accordance with the rules of Green Chemistry.

## 1. Introduction

The study of the properties and composition of plant secondary metabolites is of interest to many scientists. Among them, terpenes are a significant and very interesting group due to their comprehensive action in plants and their use in food, pharmaceutical, cosmetic or agricultural products [1]. Terpenes are isoprene oligomers (2-methylbuta-1,3-diene) usually connected together according to the isoprene rule—head-to-tail”. Monoterpenes as isoprene dimers are the simplest terpenes [2]. These compounds are the main components of essential oils found in plants of different climate zones. In plants they are located mainly in specialized types of storage tissues like glandular capitate trichomes [3,4,5]. It is surprising that their role in plants is not fully explained. So far, we know that essential oils are responsible for the fragrance of the raw material and they can attract pollinating insects [6]. Scientists have confirmed their physiological roles include chemical defenses against abiotic and biotic stresses [7,8], e.g., it protects against bacterial or fungal infection, insect or plant-eating animals. The emission of most stress-induced volatile compounds is thought to be mediated by the expression of the genes encoding the responsible proteins, such as TPS and CYPs, as well as by a burst of volatiles from storage organs when some of this organs are damaged [3]. The volatiles support the plants in the competition for habitat between different plant species (allelopathy).

Despite this designed operation, many microorganisms, insects and animals have developed a selective ability to deal with plant defense mechanisms. As a result of changes taking place in these organisms, toxic compounds are usually transformed into less toxic derivatives. These derivatives can also be useful to the organism, sometimes even more than the parent compound. For example, *Dendroctonus brevicomis* beetles feeding on *Pinus panda* dew produce two types of deterrent pheromones from the constituents of this tree’s essential oils. One of them is unchanged myrcene—the component of the essential oil, and the other is a mixture of verbenol and verbenone, which are oxidation products of α-pinene [9].

Every living organism has an enzymatic system for the elimination of potentially harmful compounds. In order to survive, it is necessary to start a detoxification process, which allows to easily removable water-soluble metabolites from the organism. There are three phases in this process. In the first phase the structure of the xenobiotic undergoes enzymatic modification (biotransformation). At this stage, the products of oxidation [10], hydroxylation [11] and hydrolysis [12] are formed. The second phase of detoxification is the coupling of the obtained compounds with polarity-enhancing molecules, such as sugars [13]. The last, third phase involves the transport of the conjugates received to the secretory organs for their excretion from the organism [12]. The phase concerns mainly insects and higher organisms [14].

Tobacco cutworm, *Spodoptera litura* (Fabricius) (Lepidoptera: Noctuidae), is a polyphagic pest, widespread throughout South and East Asia, Africa and Oceania [15]. It feeds on over 389 species of plants [16], including legumes, cotton, cabbage, cauliflower, castor, peanut, tubers and oilseeds [17]. In the states of Rajasthan and Maharashtra, India, in 2008–2009 it caused soybean losses of 64 million USD and 300 million USED respectively [18]. Such large losses are due to the fact that in humid tropics it can occur about 8 generations per year, and in India even 12 generations per year [19]. Additionally, *S. litura* is one of the 10 most resistant arthropods in the world with 638 reported cases of resistance to 39 active substances, including organophosphates, carbamates, pyrethroids and some newer chemical insecticides, such as indoxacarb, abamectin, emamectin benzoate and chlorantraniliprole [20,21]. Insect resistance to commonly used insecticides mainly involves three mechanisms such as reduced penetration, increased detoxification and target site insensitivity due to point mutations [22]. In recent years, there has also been a return to natural methods in pest control. Understanding the course of terpenes biotransformation, which is a stage of detoxification process, by these insects may be useful in design of new insecticides.

In this work we focused on the biotransformation of several popular one-ring monoterpenes. Selected compounds contain *p*-methane skeleton and are commonly found in plants and in the essential oils obtained from them. These compounds (substrates and products) have versatile biological effects and are widely used in many industries. The paper presents various biocatalysts capable of converting monoterpenes into their derivatives. As in most studies of this type [1,23], most of them are microorganisms, i.e., bacteria, yeasts and fungi. For the comparison of the type of products, in addition to microorganisms, the larvae of *Spodoptera litura*, known for their ability to biotransform terpenes, are also presented here [24].

## 2. Limonene

Limonene is a terpene unsaturated hydrocarbon. It is found in many essential oils. The (*R*)-(+) enantiomer is the main component of citrus oils, i.e., *Citrus reticulata* and *Citrus aurantium* oils. The smell of this isomer is described as citrus. On the other hand, (*S*)-(−)-limonene is present, inter alia, in *Albies alba* oil and the fragrance is described as turpentine.

### 2.1. Microbiological Biotransformation of Limonene

**Scheme 1:**
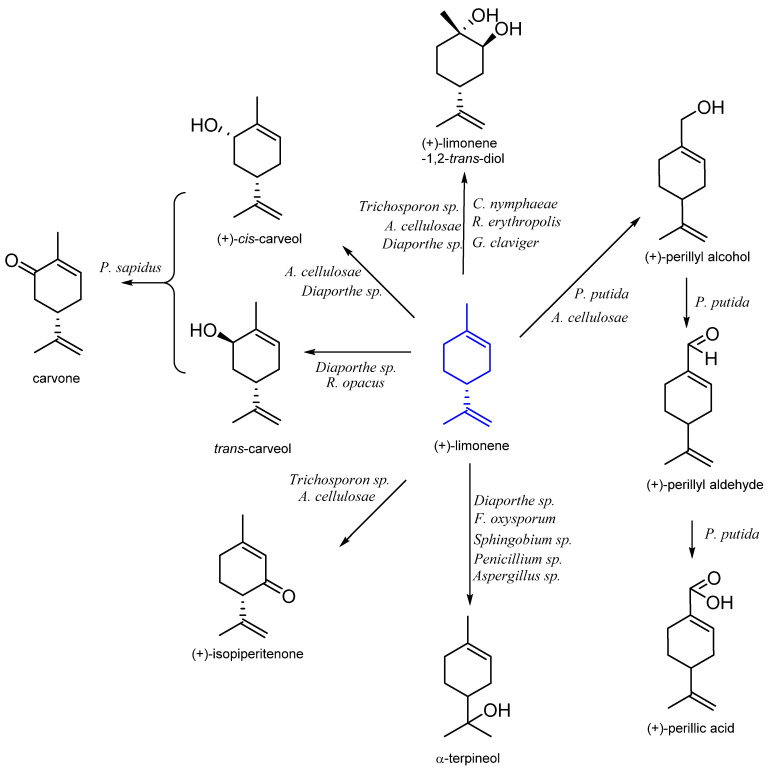
Biotransformation of (+)-limonene.

**Scheme 2:**
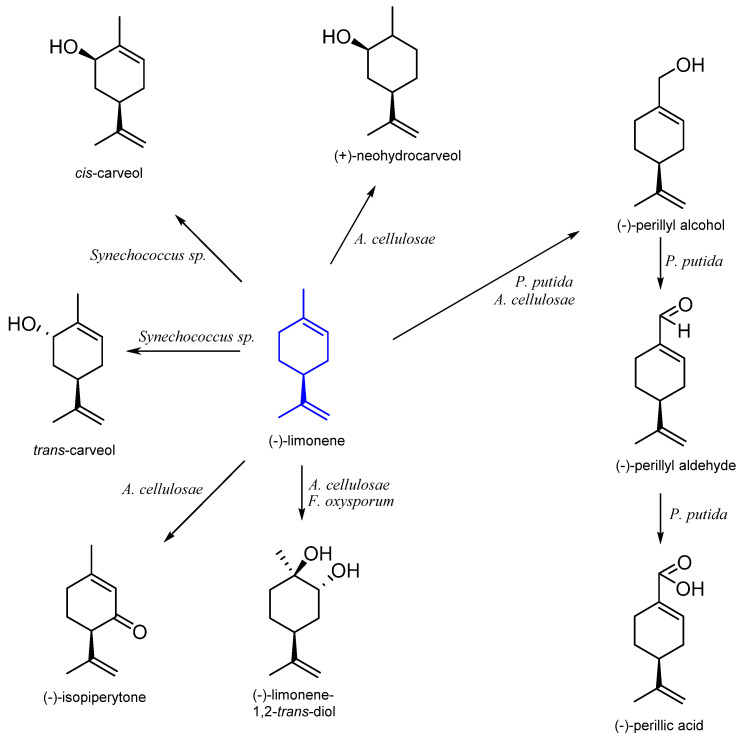
Biotransformation of (−)-limonene.

The bacteria *Pseudomonas putida* DSM 12264 is capable of converting (*R*)-(+)-limonene to (*R*)-(+)-perillic acid and (*S*)-(−)-limonene to (*S*)-(−)-perillic acid. This process involves three oxidation stages catalyzed by monooxygenase, alcohol dehydrogenase and aldehyde dehydrogenase, leading to alcohol, aldehyde and perillic acid, respectively. The highest yield (18 mM perillic acid) was obtained with the use of 150 mM limonene, 50 mM glycerol, temperature 30–34 °C and pH 7 [25]. Scheme 1 and Scheme 2, Table 1.

**Scheme 3:**
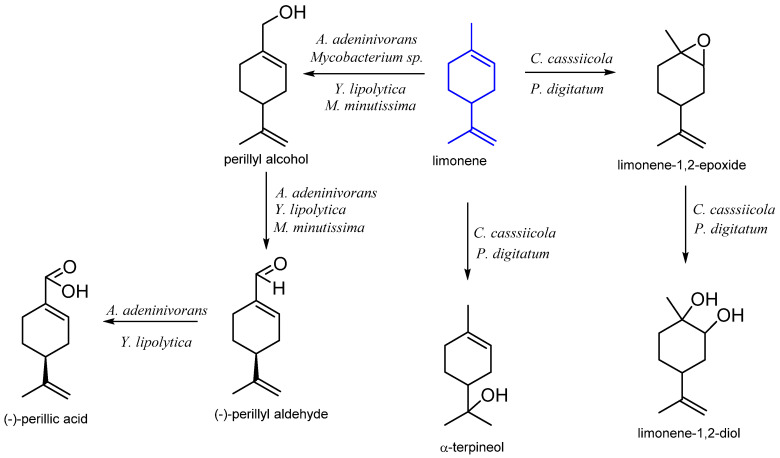
Biotransformation of racemic limonene.

On the other hand, the *Mycobacterium* sp. HXN-1500 strain carries out the oxidation of limonene to perillyl alcohol through a multi-component electron transfer chain consisting of cytochrome P450, ferredoxin and ferredoxin reductase. It was found that the ability of *Mycobacterium* sp. to hydroxylate limonene was due to its ability to hydroxylate alkanes [36]. The P450-dependent alkane monooxygenase system is responsible for the hydroxylation of alkanes. The next stage involves the action of alcohol oxidizing enzymes (alcohol oxidases or alcohol dehydrogenases) and aldehyde dehydrogenase, resulting in the formation of the appropriate acid [41]. Scheme 3, Table 1.

The yeast, in particular the strain *Yarrowia lipolytica* ATCC 18942, was able to oxidize limonene to perillic acid. The authors suggest that this process is probably initiated by monooxygenases associated with cytochrome P-450. This supposition was confirmed by an experiment in which perillyl alcohol was used as a substrate. Yeast *Y. lipolytica* was able to oxidize perillyl alcohol to perillyl aldehyde and then to perillic acid. Another experiment was carried out in the absence of oxygen, incubating the reaction mixture in a screwed-up flask in a nitrogen atmosphere. At that time, perillyl aldehyde was reduced to peril alcohol. This suggests that the oxidation of limonene to perillyl alcohol occurs in stages and through a multi-enzymatic oxidation pathway [37]. Scheme 3, Table 1.

Perillic acid was also obtained from limonene by using the yeast strains *Arxula adeninivorans* CSIR Y-1149 and *Yarrowia lipolytica* CBS 599 T [26]. Scheme 3, Table 1.

The psychrotrophic fungus *Mortierella minutissima* 01 is also capable of oxidizing C-7 carbon in the (+)-limonene molecule. As a result of the reaction carried out for 48 h at 15 °C with a substrate at 6.7 g/L, perillyl alcohol (71.5 mg/L) and perillyl aldehyde (7.9 mg/L) were obtained as main products. For additional mycelium aeration, 1% H_2_O_2_ was added to the reaction mixture. Due to the fact that the fungus *Mortierella minutissima* 01 is able to decompose H_2_O_2_ into oxygen and water via catalase, the addition of H_2_O_2_ had a positive effect on the biotransformation process, increasing its efficiency. The reaction carried out under the same conditions as described above, but with the addition of 1% hydrogen peroxide, yielded 104.9 mg/L of perillyl alcohol and 9.8 g/L of perillyl aldehyde [38]. Scheme 2, Table 1.

Common products of limonene biotransformation are *trans*-1,2-diol and α-terpineol. These compounds are formed by the use of both yeasts and filamentous fungi.

Yeast of the genus *Trichosporon* sp. UOFS Y-2041 transformed (*R*)-(+)-limonene into two products, resulting from the hydroxylation of the double bond 1,2 and the oxidation of carbon C-3. Isopiperitone was formed with an efficiency of 20% and (+)-limonene-1,2-*trans*-diol with an efficiency of 30%. During the biotransformation of (*R*)-(+)-limonene with *Trichosporon* sp. UOFS Y-0116 only isoperitone with a yield of 2% was observed [26]. Scheme 2, Table 1.

Both limonene enantiomers were subjected to biotransformation with *Aspergillus cellulosae* M-77. (*R*)-(+)-Limonene was mainly converted to (+)-limonene-1,2-*trans*-diol (21%), (+)-isopiperitenone (19%), (+)-perillyl alcohol (12%) and (+)-*cis*-carveol (5%). The biotransformation of (*S*)-(−)-limonene resulted mainly in (−)-limonene-1,2-*trans*-diol (10%), (−)-isopiperitenone (3%), (−)-perillyl alcohol (20%) and (+)-neodihydrocarveol (10%). The observed products were formed as a result of dihydroxylation of the 1,2 double bond and hydroxylation in the allylic position of C-6 and C-7 carbons. Isopiperitone was formed as a result of two successive reactions: hydroxylation of carbon C-3 and oxidation of the resulting hydroxyl group. In turn, (+)-neodihydrocarveol was formed by reducing the double bond of *trans*-carveol, present in small quantities in the post-reaction mixture. The biotransformation in the culture of *Aspergillus cellulosae* M-77 of the racemic mixture of (±)-limonene allowed obtaining 1,2-*trans*-diol, isopiperitenone, perillyl alcohol and α-terpineol [27]. Scheme 1 and Scheme 2, Table 1.

(*R*)-(+)-Limonene was subjected to solid-state fermentation with the endophytic fungus *Diaporthe* sp. Dried and ground orange waste consisting of peelings and pomace was used as substrate. The substrate was chosen due to the high content of limonene (5.08%) in peel and orange pomace. As a result of the 7-day biotransformation, limonene-1,2-diol was obtained in the amount of 2.66 g/kg of substrate. Other compounds produced in significant amounts were α-terpineol, *trans*-carveol and *cis*-carveol [28]. Scheme 2, Table 1.

Repeated tests have shown that in biotransformation of both limonene isomers it is possible to obtain α-terpineol and limonene-*trans*-diol. Various microorganisms are used for biotransformation, e.g., filamentous fungi of the genus *Fusarium oxysporum* 152B. Observations made by the authors confirmed the hypothesis that *F. oxysporum* 152B uses two parallel metabolic pathways for limonene isomers, depending on the substrate. In the case of (*S*)-(−)-limonene, the 1,2 double bond is epoxidized and the oxirane ring is opened to form the diol by an oxidation process leading to energy production. (*R*)-(+)-Limonene is converted to α-terpineol, in a process independent of oxygen, which can be seen as a process of medium detoxification [29]. Scheme 1 and Scheme 2, Table 1.

In order to optimize the biotransformation process of (*R*)-(+)-limonene to limonene-1,2-diol, the fungal strain *Colletotrichum nymphaeae* CBMAI 0864 was used. It was found that oxygenation of the medium plays an important role in biotransformation. Carrying out biotransformation in anaerobic conditions did not allow obtaining any products. The best result was obtained after addition of 15 g/L to the substrate to the mycelium growth. After 8 days of biotransformation, 4.19 g/L of limonene-1,2-diol was obtained. Scheme 2, Table 1.

The authors investigated the mechanism of diol formation. From previous studies it is known that in case of *Rhodococcus erythropolis* bacteria, in the first stage FAD- and NADH-dependent limonene-1,2-monooxygenase acts, which oxidizes limonene to limonene-1,2-epoxide, which in turn is opened to form the diol by co-factor-independent epoxide hydrolyase. *Colletotrichum nymphaeae* CBMAI 0864 strain was found to be free of these enzymes. Similarities to *Grosmannia claviger* strain were observed. In both these strains FAD-binding monooxygenase (Acc No F0 × 7A8) is responsible for the process of epoxy formation, whereas epoxide hydrolyase (Acc No F0 × 7A7) is responsible for opening the epoxide ring to form the diol [23,31]. Scheme 3, Table 1.

The use of the fungal strains *Penicillium digitatum* and *Corynespora casssicola* for biotransformation of both limonene enantiomers allowed to obtain α-terpineol and limonen-1,2-diol, respectively, as main products. The complete overreaction of limonene to α-terpineol was observed already after 8 h, while the transformation of the substrate to diol took much longer, i.e., 5 days [39]. Scheme 1 and Scheme 2, Table 1.

Orange essential oil, containing mainly (*R*)-(+)-limonene (94%), was biotransformed using strains of *Penicillium* sp. 2025, *Aspergillus* sp. 2038, *Fusarium oxysporum* 152B. In the biotransformations manioc meal (manipueira) was used as a medium. Mycelium was cultured in the manioc medium and then after growth it was transferred to the mineral medium. Orange oil was added in three portions after three, four and five days. The biotransformation was carried out for 7 days. The strain leading the process was *F. oxysporum* 152B, and the main product obtained in the amount of 450 mg/L was α-terpineol [32]. Scheme 2, Table 1.

The *Sphingobium* sp. strain was used to optimize the biotransformation of *R*-(+)-limonene to α-terpineol. The authors investigated a number of parameters such as pH, biocatalyst and substrate concentration, temperature, time and agitation. The best result, taking into account the concentration of the product, was obtained when a reaction medium was used as a mixture of water with pH = 7 and soybean oil in the proportion of 1:3, with biomass concentration of 2.8 g/L, and limonene concentration of 350 g/L. The cultivation was carried out for 96 h at 28 °C and agitation speed of 200 rpm. Under such conditions, 240 g/L of α-terpineol was obtained, which was about 20 g of a-terpineol per 1 g of biomass. On the other hand, using a mixture of water and oil in the proportion of 1:1 led obtaining α-terpineol in a lower concentration (182 g/L), but with higher yield, 65 g of α-terpineol per 1 g of biomass [30]. Scheme 2, Table 1. Limonene can also be used to make carveols. *Rhodococcus opacus* PWD4 strain, capable of degrading toluene, carried out hydroxylation of (*R*)-(+)-limonene only in the 6-position, allowing to obtain enantiomerically pure *trans*-carveol with a 97% yield. Such a result was obtained by culturing the bacteria on a mineral medium with the addition of toluene as the only carbon source. On the other hand, the bacteria grown on the medium in which the carbon source was glucose did not transform (*R*)-(+)-limonene at all. This suggests that one of the enzymes in the toluene degradation pathway, which is toluene 2,3-dioxygenase, is responsible for the biotransformation [33]. Scheme 2, Table 1.

In turn, the use of Ca^2+^-alginate-immobilized cyanobacteria *Synechococcus* sp. PCC 7942 as a biocatalyst made it possible to obtain a mixture of *cis*- and *trans*-carveols with (*S*)-(−)-limonene. After 6 h of biotransformation, 31% of *cis*-carveol and 9% of *trans*-carveol were obtained. In turn (+)-limonene did not undergo any transformations [35]. Scheme 1, Table 1.

The use of *Pleurotus sapidus* P 226-1 as a fungal biocatalyst made it possible to obtain, from (*R*)-(+)-limonene, a mixture of *cis*- and *trans*-carveols in the proportion of 2:3 and racemic carvone resulting from the oxidation of carveols [34]. Scheme 1, Table 1.

### 2.2. The Biotransformation of Limonene by Insects

**Scheme 4:**
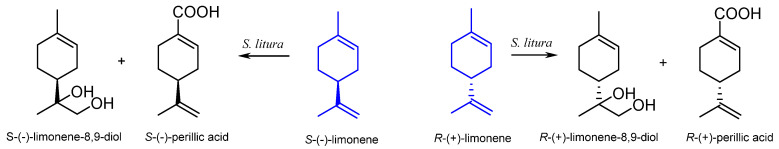
Biotransformation of (*R*)-(+)- and (*S*)-(−)-limonene by the larvae of *S. litura.*

In experiments comparing the metabolism of both enantiomers in the enzyme system of *Spodoptera litura* larvae, it has been shown that there are no great differences in the products produced and their amounts. The larvae converted both enantiomers to the corresponding limonene-8,9-diol and perillic acid (Scheme 4). These products were formed as a result of the dihydroxylation of the double bond at the 8,9 position or the oxidation of the C-7 carbon. It should be emphasized that the dihydroxylation of the 8,9 double bond is unique for insects. During the experiment, no formation of any intermediate products (alcohol, aldehyde, epoxide) was observed. Moreover, it was found that 8,9-diols arise as a mixture of diastereoisomers. On this basis, it can be concluded that the larvae do not recognize the difference between the (+)- and (−)-limonene forms [40]. Scheme 4, Table 1.

## 3. α-Terpinene 

**Scheme 5:**
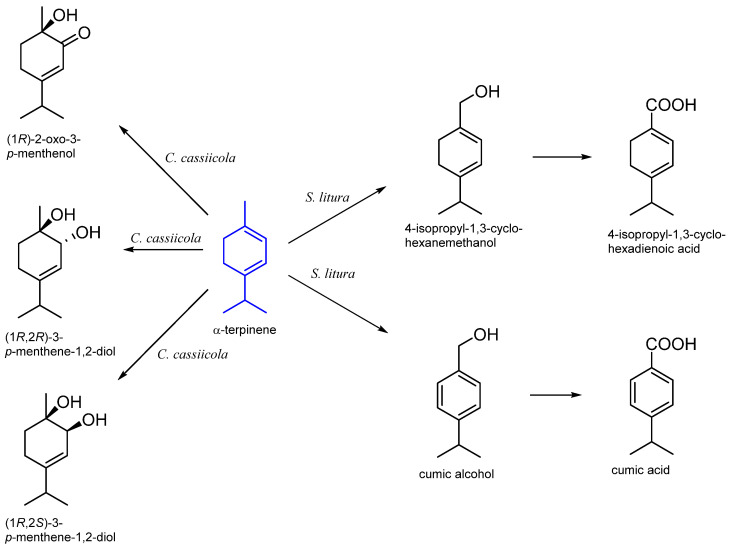
Biotransformation of racemic α-terpinene.

The fungi of the genus *Corynespora cassiicola* DSM 62475 was used for the transformation of α-terpinene. The main product of biotransformation was (1*R*,2*R*)-3-*p*-menthene-l,2-diol, obtained with a yield of 49%. The other products were (1*R*)-2-oxo-3-*p*-menthenol (2.3%) and (1*R*, 2*S*)-3-*p*-menthene-1,2-diol (1.3%) [42,43]. Scheme 5, Table 2.

α-Terpinene was given to *S. litura* Fabricius larvae in a concentration of 10 mg/g of food. As a result of biotransformation, 4-isopropyl-1,3-cyclohexadienoic acid (71.7%) and cumic acid (7.8%) were obtained as main products. Traces of intermediate products, i.e., 4-isopropyl-1,3-cyclohexadienemethanol (3.8%) and cumic alcohol (0.5%) were also observed. The examined larvae were therefore able to hydroxylate C-7 carbon and then further oxidize the obtained alcohol to acid. Further studies showed that both aerobic and anaerobic bacteria take part in α-terpine metabolism. The aerobically active intestinal bacteria are responsible for the hydroxylation of C-7 carbon, while the anaerobically active intestinal bacteria are responsible for the conversion of α-terpinene into *p*-cymene [44]. Scheme 5, Table 2. 

## 4. γ-Terpinene

γ-Terpinene is isolated from e.g., *Eucalyptus dives* oil or the terpeneol variety of marjoram oil. The scent of this compound is described as herbaceous and citrus [45].

**Scheme 6:**
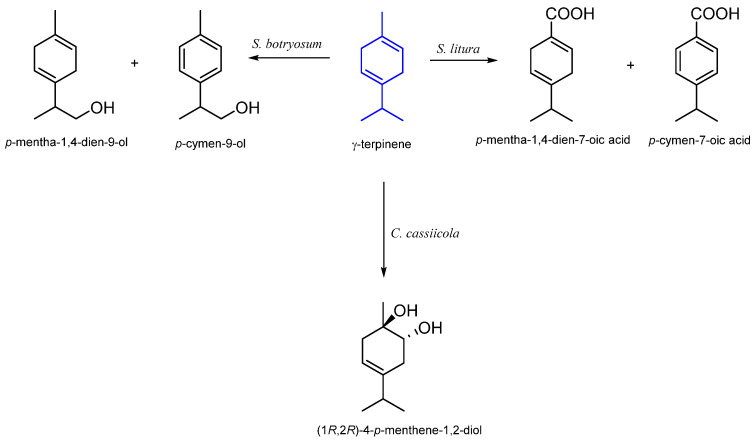
Biotransformation of racemic γ-terpinene.

Like α-terpinene, also γ-terpinene was transformed by fungi of the genus *Corynespora cassiicola* DSM 62475. In this case the main product was (1*R*,2*R*)-4-*p*-menthene-1,2-diol, obtained with an efficiency of 29% [42]. Scheme 6, Table 3.

After 24 h of culture incubation with 15 mM γ-terpinene, 60% *p*-mentha-1,4-dien-9-ol and 20% *p*-cymen-9-ol were obtained. The process was highly regio- and enantioselective, because introduction of hydroxyl group to inactivated C9 in γ-terpinene allowed to obtain alcohols with ee = 74% for *p*-mentha-1,4-dien-9-ol and ee = 70% for *p*-cymen-9-ol. It is worth noting that *p*-mentha-1,4-dien-9-ol showed a characteristic herbaceous smell, resembling dill, with a flavor threshold of 5 μg in air. The formation of *p*-cymen-9-ol is most likely due to the transformation of γ-terpinene into cymene, due to the aromatization of the cyclohexene ring. The resulting cymene is then hydroxylated [46]. Scheme 6, Table 3.

Interestingly, when the strain *S. botryosum* from the DSMZ collection (*S. botryosum* DSMZ 62928) was used to transform γ-terpinene, only 4% *p*-mentha-1,4-dien-9-ol was obtained. The use of *Thelebolus caninus* CBS 710.69 for biotransformation of the same substrate resulted in 15.7% of *p*-mentha-1,4-dien-9-ol [46]. Scheme 6, Table 3.

As a result of administration of γ-terpinene at a concentration of 1 mg/g of food for *S. litura* larvae, two main products were obtained. These were *p*-mentha-1,4-dien-7-oic acid (46%) and *p*-cymen-7-oic acid (48%). Similarly, as in the case of α-terpineol, oxidation of C-7 carbon was observed here. However, unlike then, intestinal bacteria were not involved in the above process. This difference may be due to a slightly different substrate structure and the use of a different substrate concentration in the insect diet [47]. Scheme 6, Table 3.

## 5. Terpinen-4-ol

**Scheme 7:**
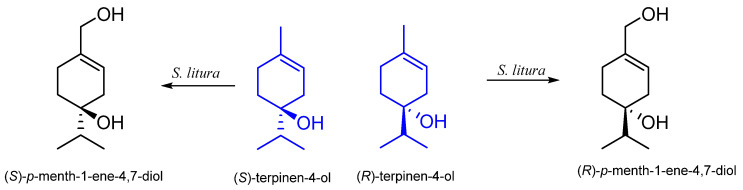
Bioransformation of (*R*)- and (*S*)-terpinen-4-ols by the larvae of *S. litura.*

Both enantiomers and racemic terpinen-4-ol are found in many essential oils, such as lavender, eucalyptus or pine. The smell is described as spicy, nut-mag, wood-earthy with a distinct lilac-like note. This compound is used in perfumery when creating tea and lavender notes.

(*R*)-Terpinen-4-ol and (*S*)-terpinen-4-ol were subjected to biotransformation in the enzymatic system of *S. litura* larvae. These compounds were given to insects at a concentration of 1 mg/g of food. Each of the enantiomers was converted to one metabolite, respectively (*R*)-*p*-menth-1-ene-4,7-diol (71%) and (*S*)-*p*-menth-1-ene-4,7-diol (72%). In both substrates C-7 carbon was hydroxylated. Moreover, the larvae did not differentiate the form of (*R*) and (*S*) of the substrate, i.e., the asymmetric carbon atom at C-4 did not affect the course of the reaction. It was also found that intestinal bacteria did not participate in the observed transformations [47]. Scheme 7, Table 4.

## 6. α-Terpineol

**Scheme 8:**
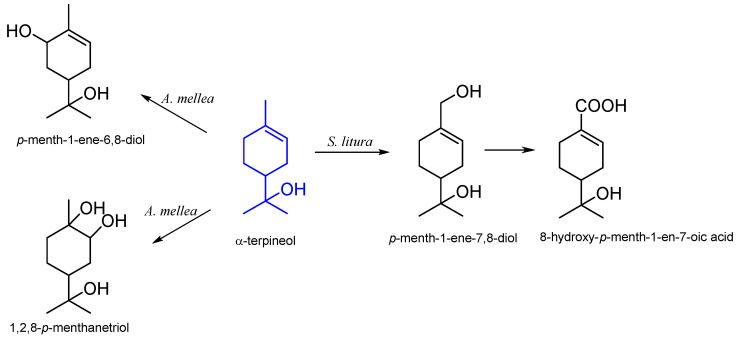
Biotransformation of racemic α-terpineol.

**Scheme 9:**
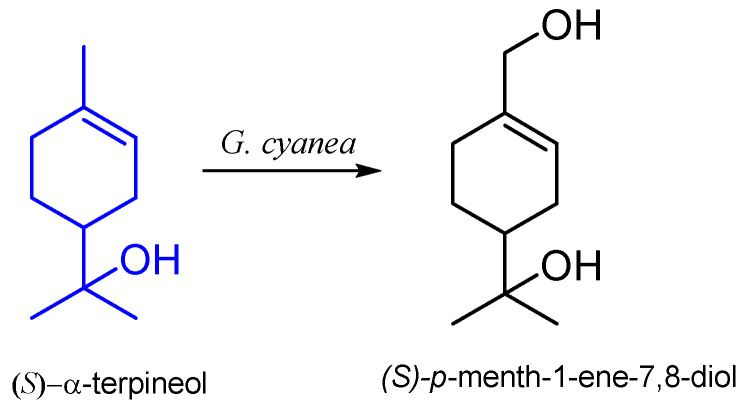
Biotransformation of (S)- α-terpineol.

This compound exists as a colorless crystalline solid. The scent is determined as lilac-like. Due to its fragrance properties, it is used as a fragrance.

(±)-α-Terpineol was biotransformed in the culture of *Armillariella mellea*. In this case two types of reactions were observed: hydroxylation in allylic position and dihydroxylation of double bond 1,2. In both cases the isomer (+) reacted faster than the isomer (−) [42,48]. Scheme 8, Table 5.

(*S*)-(−)- α-Terpineol was subjected to biotransformation in the culture of *Gibberella cyanea* DSM 62719. The main reaction product was (4*S*)-*p*-menthene-7,8-diol (33%) [42]. Scheme 9, Table 5.

*S. litura* larvae were administered (±)-α-terpineol in an amount of 10 mg per g of insect body weight on three consecutive days. As a result of biotransformation, two products were obtained, i.e., *p*-menth-1-ene-7,8-diol (26.7%) and 8-hydroxy-*p*-menth-1-en-7-oic acid (57.6%). Analysis of the results showed that the C-7 carbon was hydroxylated in the first step, followed by the primary alcohol oxidation to acid in the next step. Additionally, the conducted studies showed that intestinal bacteria were not involved in these transformations [49,50]. Scheme 8, Table 5.

## 7. Menthol

Menthol has there chiral centers; therefore, four pairs of enantiomers are known. Only (−)-menthol has a pure mint scent and it is found in many essential oils, mainly of the *Mentha* genus.

### 7.1. Microbiological Biotransformation of Menthol

**Scheme 10:**
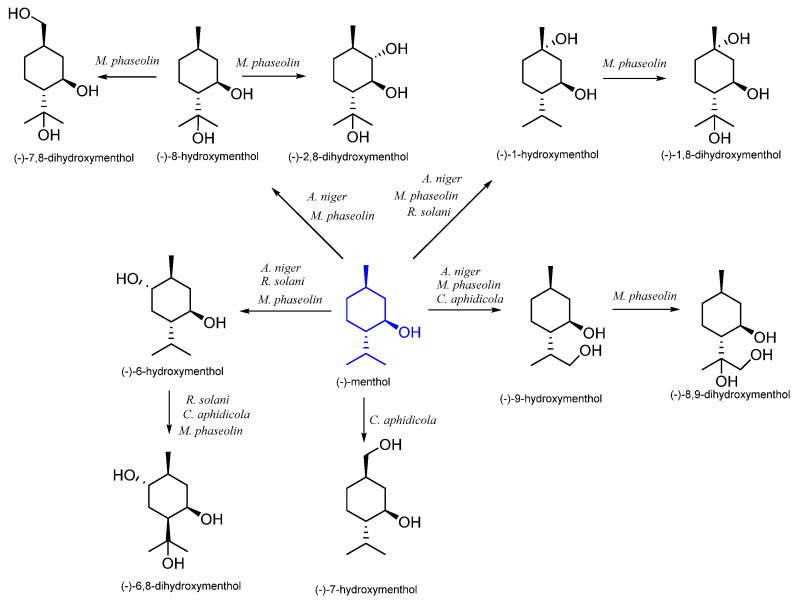
Microbial biotransformations of (−)-menthol.

**Scheme 11:**
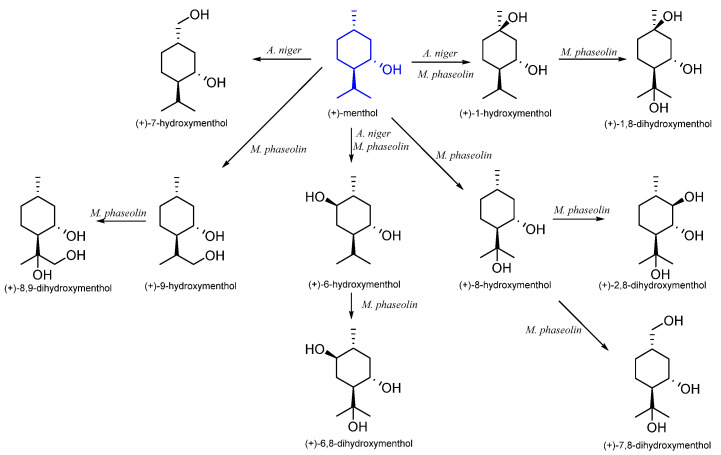
Microbiological biotransformations of (+)-menthol.

The use of fungi of the genus *Aspergillus niger* for the biotransformation of (1*R*,3*S*,4*R*)-(−)- and (1*S*,3*R*,4*S*)-(+)-menthol allowed obtaining several different hydroxyl derivatives. Biotransformations were carried out in static and shaking culture for 3 days, which allowed for the complete reactivation of the substrates. For (−)-menthol the preferred sites for hydroxylation were carbons C-8 and C-9, while for (+)-menthol it was carbon C-7. Moreover, in both cases formed were (in small amounts) hydroxylation products at C-6 and C-1 positions. Scheme 10 and Scheme 11, Table 6.

This means that the structure of the molecule has a great influence on the site of hydroxylation. As a result of the biotransformations, 20% of (−)-8-hydroxymenthol, 22% of (−)-9-hydroxymenthol, 12% of (−)-6-hydroxymenthol and 7% of (−)-1-hydroxymenthol were obtained. In the case of (+)-menthol derivatives, mainly (+)-7-hydroxymenthol (52%) and (+)-6-hydroxymenthol (6%) and (+)-1-hydroxymenthol (14%) were obtained [51]. Scheme 10 and Scheme 11, Table 6.

The fungi of the genus *Cephalosporium aphidicola* were used for the biotransformation of (−)-menthol. As a result of the 12-day incubation, several dihydroxy derivatives were obtained. These fungi preferred primarily the hydroxylation of C-7 and C-9, and, to a lesser extent, also C-6 and C-8 [52]. Scheme 10, Table 6.

For (−)-menthol biotransformation 12 isolates from *Rhizoctonia solani*, a plant pathogen commonly found in soil, were used. These isolates were derived from infected rice, Kentucky blue grass, white clover, European pear, sugar beet and coffee. Three of them, which came from sugar beet, were able to transform the substrate into products with a yield of 89.7–99.9% within 5 days. The metabolic pathways of (−)-menthol biotransformation by *Rhizoctonia solani* were studied. It was found that in the first stage there was stereoselective hydroxylation in C-1 or C-6 position. In the second stage (−)-hydroxymenthol was hydroxylated in C-8 position [53]. Scheme 10, Table 6.

Incubation of the pathogenic fungus *Macrophomina phaseolin* with (+)-menthol within 12 days allowed obtaining products resulting from hydroxylation of C-1, C-6, C-8 and C-9 carbon. These compounds were then subjected to another hydroxylation giving further products, with C-8 carbon being the preferred hydroxylation position [55]. Scheme 11, Table 6.

**Scheme 12:**
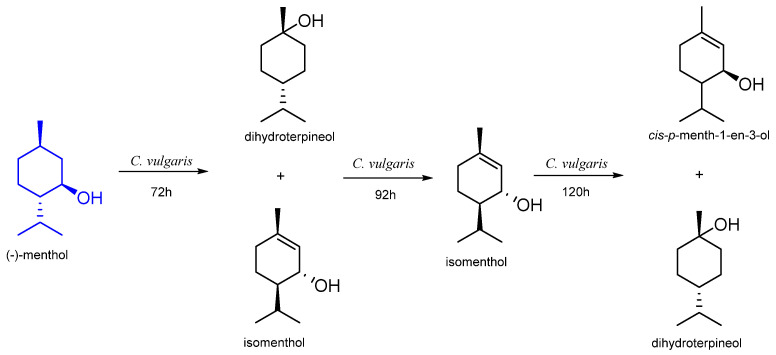
Biotransformation of (−)-menthol by microalgae.

The use of *Chlorella vulgaris* microalgae for the bioconversion of menthol resulted in obtaining various products that appeared as the reaction progressed. After 72 h of biotransformation, the appearance of dihydroterpineol (48.8%) and isomenthol (20.2%) was observed. After 92 h, the major product in the reaction mixture was isomenthol (92.3%). After 120 h of experimentation, the appearance of *cis*-*p*-menth-1-en-3-ol (46.0%) and dihydroterpineol (49.2%) was observed. The optimal pH value for this process was 5.5. The authors explained the formation of dihydroterpineol from menthol by the formation of carbocation as an intermediate product, and then its rearrangement [56]. Scheme 12, Table 6.

### 7.2. The Biotransformation of Menthol by Insects

**Scheme 13:**
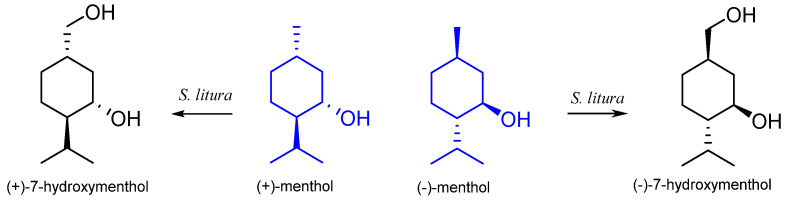
Biotransformation of menthol by insects.

(1*R*,3*S*,4*R*)-(−)-Menthol and (1*S*,3*R*,4*S*)-(+) menthol were fed to *Spodoptera litura* larvae in the diet at a concentration of 1 mg of compound per gram of food. Carbon C-7 oxidation products were obtained from both menthol isomers. From (−)-menthol obtained was (−)-7-hydroxymenthol, and from (+)-menthol analogously (+)-7-hydroxymenthol was obtained. The percentage of substrate conversion in both cases was similar and amounted to 86–90%. The process of menthol biotransformation in an in vitro system in the culture of intestinal bacteria of *Spodoptera litura* larvae was also investigated. However, it turned out that they were not involved in the metabolism of this substrate [54]. Scheme 13, Table 6.

## 8. Menthone

Menthone is an example of a terpenoid that contains a carbonyl group. This compound accompanies menthol and limonene in peppermint oils. It is used in the food industry as a flavoring ingredient, as well as in fragrance compositions.

### 8.1. Microbiological Biotransformation of Menthone

**Scheme 14:**
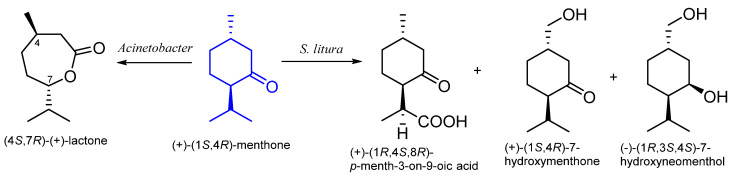
Biotransformation of (1*S*,4*R*)-(+)-menthone.

The bacterial strains *Acinetobacter* NCIEI 9871 and *Acinetobacter* TD63 turned out to be able to transform menthone in a completely different way from the ones previously encountered. These bacteria, instead of reducing the carbonyl group, carried out the Baeyer-Villiger oxidation, yielding a lactone product. The biotransformation efficiency was 90% for the *Acinetobacter* NCIEI 9871 strain and 61% for the *Acinetobacter* TD63 strain. The mentioned bacterial strains showed high substrate specificity. They oxidized only the (+)-menthone enantiomer while the (−)-enantiomer remained intact in the reaction mixture. This substrate specificity is related to the Baeyer–Villiger reaction mechanism, which involves the nucleophilic attack of the ketone by hydroperoxyflavin, leading to the formation of an intermediate hydroxyperoxyflavine. According to the authors, the location of the intermediate compound in the active site of the enzyme, as well as certain stereoelectronic effects determine the regioselectivity, and thus the enantioselectivity of the reaction. The comparison of various peroxide forms that can arise as intermediate forms allowed stating that in order to match the active site of the enzyme, the isopropyl group must be in the equatorial position. The axial orientation of the propyl group makes it impossible to match the intermediate product to the active center of the enzyme due to steric hindrance [57]. Scheme 14, Table 7.

Another microorganism capable of stereoselective reduction of the carbonyl group in the (1*R*,4*S*)-(−)-menthone molecule was the yeast strain *Hormonema* sp. UOFS Y-0067. The reaction product was (1*R*,3*S*,4*S*)-(+)-neomenthol [59]. Scheme 14, Table 7.

**Scheme 15:**
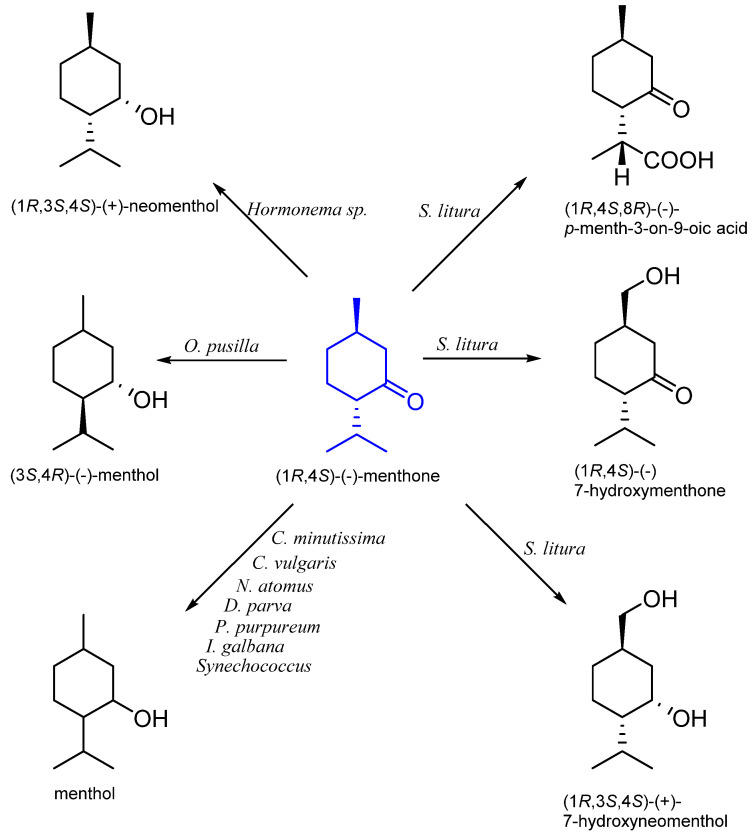
Biotransformation of (1*R*,4*S*)-(‒)-menthone.

The (1*R*,4*S*)-(−)-menthone isomer was used as a substrate for the microalgae cultures of *Chlorella minutissima*, *Nannochloris atomus*, *Dunaliella parva*, *Porphyridium purpureum* and *Isochrysis galbana*. All algae showed the ability to carry out non-stereoselective reduction of the carbonyl group. After 5 days of biotransformation, only (1*RS*,3*S*,4*R*)-(−)-menthol was present in the post-reaction mixture in addition to the substrate. *I. galbana* algae showed the highest degree of transformation, in the culture of which 37% menthol was obtained [60]. Scheme 15, Table 7.

The same substrate, (1*R*, 4*S*)-(−)-menthone, was subjected to 24 h biotransformation in *Oocystis pusilla* microalgae culture. These algae is also able to reduce the carbonyl group, yielding (1*R*,3*S*,4*R*)-(−)-menthol as a product in a yield of 11% [61]. As a result of subjecting (1*R*,4*S*)-(−)-menthone to biotransformations in the culture of *Chlorella vulgaris* MCCS 012 algae, menthol was obtained in the form of a racemate with a yield of 43% [62]. A similar reaction was observed for the cyanobacteria *Synechococcus* PCC 6716. It was also found that the cyanobacteria strains *Synechococcus* 6911 and *Anabaena oscillarioides* were not able to reduce menthone [63]. Scheme 15, Table 7.

### 8.2. The Biotransformation of Menthone by Insects

*Spodoptera litura* larvae have been tested for the biotransformation capacity of (1*R*,4*S*)-(−)-menthone and (1*S*,4*R*)-(+)-menthone. As a result of the conducted experiments, three derivatives were obtained. The main product of the (1*R*,4*S*)-(−)-menthone biotransformation was *p*-menth-3-on-9-oic acid (48.6%). The next product was 7-hydroxymenthone (20.7%) resulting from the hydroxylation of carbon C-7. This compound was reduced by the carbonyl group to give 7-hydroxyneomenthol (22.3%). For (1*S*,4*R*)-(+)-menthone, the formation of analogous products with the (+)-configuration was observed in the amounts of 46.1%, 25.0% and 20.1%, respectively. It was found that the oxidation of C-9 carbon is a reaction characteristic only for the tested insects, not previously observed. The remaining products, i.e., 7-hydroxymenthone and 7-hydroxyneomenthol, are known products, often found in nature [58]. Scheme 14, Table 7.

## 9. Carvone

The optical isomers of this compound differ in terms of fragrance properties. (+)-Carvone has herbaceous odor which is reminiscent to caraway and dill seeds and occurs in caraway and dill oil. (−)-Carvone has herbaceous odor with a note of spearmint and occurs in spearmint oil.

### 9.1. Microbiological Biotransformation of Carvone

**Scheme 16:**
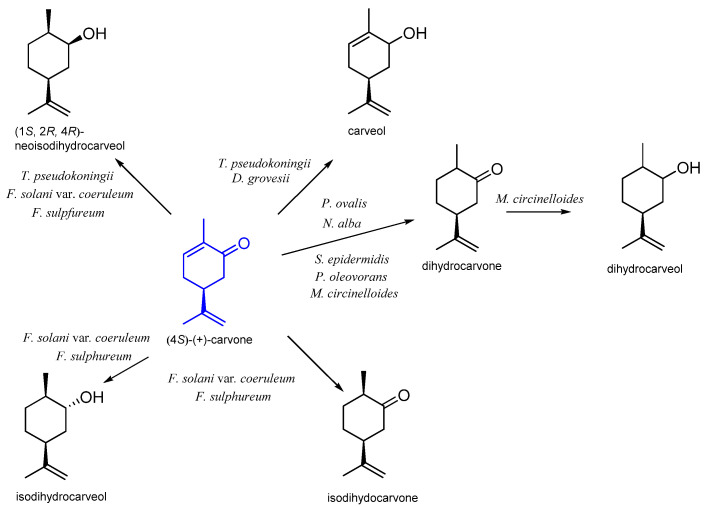
Biotransformations of (4*S*)-(+)-carvone.

In microbial biotransformations of a carvone, the most frequently observed reaction is the reduction of the double bond followed by the reduction of the carbonyl group [64]. In studies conducted by Verstegen-Haaksma [65] (4*S*)-(+)-carvone was reduced to both dihydrocarvones in the bacterial cultures. Scheme 16, Table 8.

The highest stereoselectivity of the reduction of the double bond was observed in the case of *Pseudomonas ovalis*, where the (1*S*,4*S*)-dihydrocarvone was obtained with 93% diastereomeric excess. In the culture of *Trichoderma pseudokoningii*, a different course of transformation was observed, because on the one hand there was a reduction of the carbonyl group of the carvone, leading to the formation of both carveols, and on the other hand, neoiso-dihydrocarveol was formed with an efficiency of 71%. The toxic effect of the substrate limited yields in batch-cultures is 0.2 g/L [65]. On the other hand, in the case of the transformation of (4*S*)-(+)-carvone by *Fusarium sulphureum* and *F. solani* var. *coeruleum* mainly formed were isodihydrocarvone, isodihydrocarveol and neoiso-dihydrocarveol [66]. Scheme 16, Table 8.

In the culture of *Diplogelasinospora grovesii* IMI 171018 (4*S*)-(+)-carvone was reduced to a mixture of carveol with a predominance of 2*R* isomer, without breaking the double bond in the ring [67]. During the (4*S*)-(+)-carvone biotransformation, which was carried out in the *Mucor circinelloides* culture, 75% (1*R*,4*S*)-dihydrocarvone was formed after 4 h of transformation and in smaller quantities a second diastereoisomeric dihydrocarvone and dihydrocarveols were formed. As a result of the reduction of endocyclic double carvone bond catalyzed by enone reductase, both dihydrocarvone isomers were formed in a 9:1 ratio. The reduction of the carbonyl dihydrocarvone group was already without stereoselectivity. It is worth noting that the *Mucor* strain used was isolated from *Pinus taeda*, a plant that is a good producer of monoterpenoids, including carvone [68]. Scheme 16, Table 8.

**Scheme 17:**
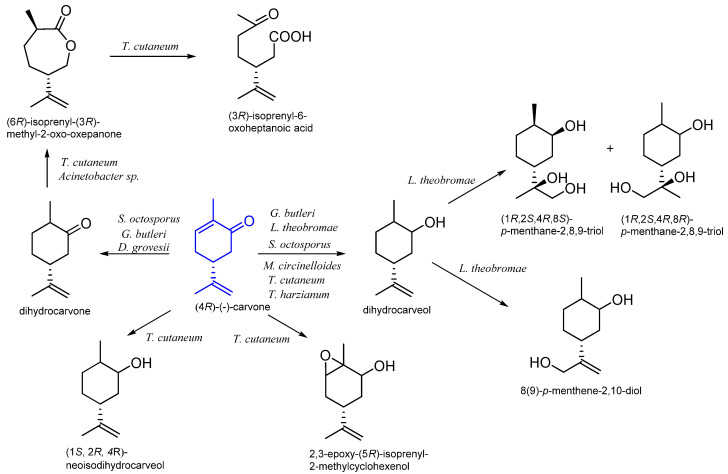
Biotransformations of (4*R*)-(−)-carvone.

In the same culture, *M. circinelloides* also underwent a transformation of (4*R*)-(−)-carvone and additional formation of trihydroxylated menthanetriols was observed by changing the extraction solvent from ethyl acetate to *n*-butanol. The first stage of biotransformation was a stereo-selective reduction of double bond and carbonyl groups catalyzed by enone reductase and carbonyl reductase respectively. The next step was dihydroxylation of the remaining double bond, leading to trihydroxylated mentanotriols. Since in the proposed pathway of biotransformation dihydrocarveol is a biosynthetic precursor of mentanotriols, stereogenic centers on C-1 and C-2 in dihydrocarveol are not involved in the biotransformation process. For this reason, both mentanotriols and dihydrocarveol must have the same absolute configuration. (1*R*,2*S*,4*R*,8*S*)-*p*-menthane-2,8,9-triol was also formed during biotransformation in the *Lasiodiplodia theobromae* BRF118 culture. The same authors also described the biotransformation of (4*R*)-(−)-carvone in the culture of *Trichoderma harzianum* BRF117, where the only isolated product was neodihydrocarveol [68]. Scheme 17, Table 8.

In the culture of *D. grovesii* IMI 171018 (4*R*)-(−)-carvone was reduced to (1*R*,2*S*,4*R*)-dihydrocarveol (90%) and (1*R*,4*R*)-dihydrocarvone (9%) [67]. The same authors searched a library of 416 strains for their bioreduction capacity, their tolerance to high ketone concentrations, and the absence of by-products in the reduction of cycloalkanones. A further 2 strains were selected: *Gongronella butleri* CBS 157.25 and *Schizosaccharomyces octosporus* NCYC 427. (4*R*)-Carvone was converted to (1*R*,4*R*)-dihydrocarvone, which was then reduced to (1*R*,2*S*,4*R*)-dihydrocarveol [69]. Scheme 17, Table 8.

A different course of transformation (4*R*)-(−)-carvone was observed in the culture of *Trichosporum cutaneum* CCT 1903. Lactone was produced (31%) after reduction of the double bond in the substrate molecule. Additional products were (3*R*)-isopropenyl-6-oxoheptanoic acid, dihydrocarveol and epoxydihydrocarveol [70]. Scheme 17, Table 8.

The lactone (6*R*)-isoprenyl-(3*R*)-methyl-2-oxo-oxepanone was formed by Baeyer–Villiger monooxygenase (BVMO). The formation of lactone is the first stage of degradation of alicyclic monoterpenes and the next stage is the opening of the lactone ring which produces ketoacid [57]. Scheme 17, Table 8.

Baeyer-Villiger oxidation of the dihydrocarone was also observed in the culture of *Acinetobacter* sp. NCIB 9871 and *Acinetobacter* sp. TD63 [57,72]. (+)-Dihydrocarvone was converted to a lactone in which the carbonyl group is next to the methyl group due to the higher migration capacity of the tertiary carbon atom adjacent to the carbonyl group. On the other hand, with (−)-dihydrocarvone, a lactone was formed in both cultures, in which the oxygen atom is located between the methyl and carbonyl groups. The resulting lactone is an intermediate in the synthesis of (3*S*,6*R*)-3-methyl-6-(1-methylethenyl)-9-decen-1-yl acetate, which is an attractant for male *Aonidiella aurantii*—a citrus pest and can be used in combination with other agents for controlling these pests [73]. Scheme 17, Table 8.

### 9.2. The Biotransformation of Carvone by Insects

**Scheme 18:**
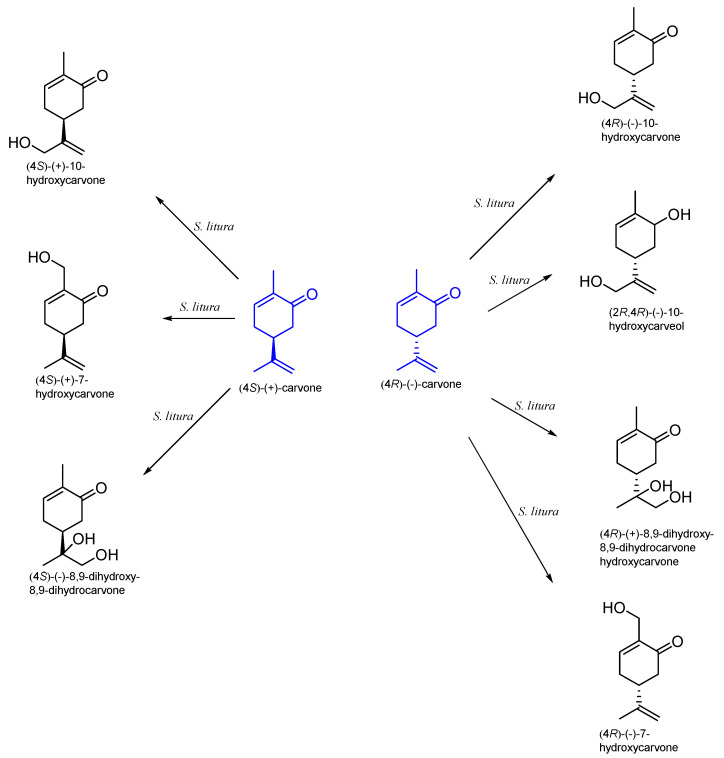
Biotransformation of (4*S*)-(+)-carvone and (4*R*)-(−)-carvone by insects.

The metabolism of both carvone stereoisomers was investigated in *Spodoptera litura* [71]. The substrate was administered to the larvae at a concentration of 1 mg/g of diet. Furthermore, in this case, hydroxylation reactions were observed. During the biotransformation of the (4*S*)-(+)-carvone, three compounds were formed. The result was 27% of (4*S*)-(+)-10-hydroxycarvone, 11% of (4*S*)-(+)-7-hydroxycarvone, and 48% of (4*S*)-(−)-8,9-dihydroxy-8,9-dihydrocarvone. Scheme 18, Table 8.

In turn, during biotransformation of (4*R*)-(−)-carvone obtained were four hydroxyl derivatives: 17% of (4*R*)-(−)-10-hydroxycarvone, 8% of (4*R*)-(−)-7-hydroxycarvone, 45% of (4*R*)-(+)-8,9-dihydroxy-8,9-dihydrocarvone, and 20% of (2*R*,4*R*)-(−)-10-hydroxycarveol. There is a difference in the process of transformation between carvone isomers because only in the case of (4*R*)-(−)-carvone, the carbonyl group in the product of hydroxylation on C-10 carbon: (4*R*)-(−)-10-hydroxycarvone was then reduced stereoselectively to (2*R*,4*R*)-(−)-10-hydroxycarveol. It is noteworthy that in all obtained derivatives the unsaturated carbon-carbon double bond in the ring remained intact.

## 10. Conclusions

Research on the transformation of terpene compounds in the enzymatic systems of living organisms has been conducted since the mid-twentieth century. Bacteria, fungi and insects catalyze several specific reactions that make it possible to obtain derivatives that are very difficult to obtain with standard organic synthesis. The reactions that give products with high enantiomeric excess or with specific regioselectivity are worth mentioning here. The use of an enzyme bouquet of living organisms allows us to reduce the production costs of biologically active compounds and reduce possible environmental pollution.

The review indicated that monoterpenes with *p*-methane system are very interesting substrates for biotransformation with enzymatic system of microorganisms and insects. From the information we have gathered, it appears that the formation of oxidation products is mainly preferred. Such reaction products were described in manuscripts on transformations of both hydrocarbon, alcohol and ketone compounds. A variety of very interesting derivatives was obtained depending on the biocatalyst used. Some of them are very important for their application in perfumery, cosmetics, food and pharmaceutical industries e.g., menthol, terpinen-4-ol [74,75,76,77,78].

## Figures and Tables

**Table 1:** Biotransformation of limonene.

Stereoisomer	Organism	Products	Reference
(*R*)-(+)-	*Pseudomonas putida* DSM 12264	(*R*)-(+)-perillic acid	[25]
	*Trichosporon* sp. UOFS Y-2041	isopiperitone (+)-limonene-1,2-*trans*-diol	[26]
	*Trichosporon* sp. UOFS Y-0116	isopiperitone	
	*Aspergillus cellulosae* M-77	(+)-limonene-1,2-*trans*-diol (+)-isopiperitenone (+)-perillyl alcohol (+)-*cis*-carveol	[27]
	*Diaporthe* sp.	limonene-1,2-diol α-terpineol carveol	[28]
	*Fusarium oxysporum* 152B	α-terpineol	[29]
	*Sphingobium* sp.	α-terpineol	[30]
	*Colletotrichum nymphaeae* CBMAI 0864 *Rhodococcus erythropolis* *Grosmannia claviger*	limonene-1,2-epoxide limonene-1,2-diol	[31]
	*Penicillium* sp. 2025 *Aspergillus* sp.2038 *Fusarium oxysporum* 152B	α-terpineol	[32]
	*Rhodococcus opacus* PWD4	carveol	[33]
	*Pleurotus sapidus* P 226-1	carveol carvone	[34]
(*S*)-(−)	*Pseudomonas putida* DSM 12264	(*S*)-(-)-perillic acid	[25]
	*Aspergillus cellulosae* M-77	(−)-limonene-1,2-*trans*-diol (−)-isopiperitenone (−)-perillyl alcohol (+)-neodihydrocarveol	[27]
	*Fusarium oxysporum* 152B	limonene-1,2-epoxide limonene-1,2-diol	[29]
	*Synechococcus* sp. PCC 7942	carveol	[35]
n. d.	*Mycobacterium* sp. HXN-1500	perillyl alcohol	[36]
	*Yarrowia lipolytica* ATCC 18942	perillic acid	[37]
	*Arxula adeninivorans* CSIR Y-1149 *Yarrowia lipolytica* CBS 599 T	perillic acid	[26]
	*Mortierella minutissima* 01	perillyl alcohol perillyl aldehyde	[38]
	*Penicillium digitatum* *Corynespora casssicola*	α-terpineol limonene-1,2-diol	[39]
	*Spodoptera litura*	limonene-8,9-diol perillic acid	[40]

**Table 2:** Biotransformation of α-terpinene.

Organism	Products	Reference
*Corynespora cassiicola* DSM 62475	3-*p*-menthene-l,2-diol 2-oxo-3-*p*-menthenol 3-*p*-menthene-1,2-diol	[42,43]
*Spodoptera litura*	4-isopropyl-1,3-cyclohexadienemethanol 4-isopropyl-1,3-cyclohexadienoic acid cumic alcohol cumic acid	[44]

**Table 3:** Biotransformation of γ-terpinene.

Organism	Products	Reference
*Corynespora cassiicola* DSM 62475	4-*p*-menthene-1,2-diol	[42]
*Stemphylium botryosum*	*p*-mentha-1,4-dien-9-ol *p*-cymen-9-ol	[46]
*S. botryosum* DSMZ 62928	*p*-mentha-1,4-dien-9-ol	
*Spodoptera litura*	*p*-mentha-1,4-dien-7-oic acid *p*-cymen-7-oic acid	[47]

**Table 4:** Biotransformation of terpinen-4-ol.

Organism	Products	Reference
*Spodoptera litura*	*p*-menth-1-ene-4,7-diol	[47]

**Table 5:** Biotransformation of α-terpineol.

Stereoisomer	Organism	Products	Reference
n. d.	*Armillariella mellea*	*p*-menthene-6,8-diol *p*-menthene-1,2,8-triol	[42,48]
(*S*)-(−)	*Gibberella cyanea* DSM 62719	*p*-menthene-7,8-diol	[42]
n. d.	*Spodoptera litura*	*p*-menth-1-ene-7,8-diol 8-hydroxy-*p*-menth-1-en-7-oic acid	[49,50]

**Table 6:** Biotransformation of menthol.

Stereoisomer	Organism	Products	Reference
(1*R*,3*S*,4*R*)-(−)-	*Aspergillus niger*	(−)-1-hydroxymenthol (−)-6-hydroxymenthol (−)-8-hydroxymenthol (−)-9-hydroxymenthol	[51]
	*Cephalosporium aphidicola*	(−)-6-hydroxymenthol (−)-7-hydroxymenthol (−)-8-hydroxymenthol (−)-9-hydroxymenthol	[52]
	*Rhizoctonia solani*	(−)-1-hydroxymenthol (−)-6-hydroxymenthol (−)-6,8-dihydroxymenthol	[53]
	*Spodoptera litura*	(−)-7-hydroxymenthol	[54]
(1*S*,3*R*,4*S*)-(+)-	*Aspergillus niger*	(+)-1-hydroxymenthol (+)-6-hydroxymenthol (+)-7-hydroxymenthol	[51]
	*Macrophomina phaseolin*	(+)-1-hydroxymenthol (+)-6-hydroxymenthol (+)-8-hydroxymenthol (+)-9-hydroxymenthol (+)-1,8-dihydroxymenthol (+)-2,8-dihydroxymenthol (+)-6,8-dihydroxymenthol (+)-7,8-dihydroxymenthol (+)-8,9-dihydroxymenthol	[55]
	*Spodoptera litura*	(+)-7-hydroxymenthol	[54]
n. d.	*Chlorella vulgaris*	dihydroterpineol isomenthol *cis*-*p*-menth-1-en-3-ol	[56]

**Table 7:** Biotransformation of menthone.

Stereoisomer	Organism	Products	Reference
(1*S*,4*R*)-(+)-	*Acinetobacter* NCIEI 9871*Acinetobacter* TD63	(4*S*,7*R*)-(+)-mentholactone	[57]
	*Spodoptera litura*	7-hydroxymenthone 7-hydroxyneomenthol *p*-menth-3-on-9-oic acid	[58]
(1*R*,4*S*)-(−)-	*Hormonema* sp. UOFS Y-0067	neomenthol	[59]
	*Chlorella minutissima* *Nannochloris atomus* *Dunaliella parva* *Porphyridium purpureum* *Isochrysis galbana*	menthol	[60]
	*Oocystis pusilla*		[61]
	*Chlorella vulgaris* MCCS 012		[62]
	*Synechococcus* PCC 6716		[63]
	*Spodoptera litura*	7-hydroxymenthone 7-hydroxyneomenthol *p*-menth-3-on-9-oic acid	[58]

**Table 8:** Biotransformation of carvone.

Stereoisomer	Organism	Products	Reference
(*S*)-(+)	*Pseudomonas ovalis*	dihydrocarvone	[65]
	*Staphylococcus epidermidis*		
	*Nocardia alba*		
	*Pseudomonas oleovorans*		
	*Trichodema pseudokoningii*	carveol neoiso-dihydrocarveol	
	*Fusarium sulphureum*	isodihydrocarvone isodihydrocarveol neoiso-dihydrocarveol	[66]
	*F. solani* var. *coeruleum*		
	*Diplogelasinospora grovesii* IMI 171018	carveol	[67]
	*Mucor circinelloides*	dihydrocarvone dihydrocarveol	[68]
	*Spodoptera litura*	(4*S*)-(+)-10-hydroxycarvone (4*S*)-(+)-7-hydroxycarvone 8,9-dihydroxy-8,9-dihydrocarvone	[58]
(*R*)-(−)	*Mucor circinelloides*	dihydrocarvone dihydrocarveol *p*-menthane-2,8,9-triol	[68]
	*Lasiodiplodia theobromae* BRF118	(1*R*,2*S*,4*R*,8*S*)-*p*-menthane-2,8,9-triol	
	*Trichoderma harzianum* BRF117	neodihydrocarveol	
	*Diplogelasinospora grovesii* IMI 171018 *Gongronella butleri* CBS 157.25 *Schizosaccharomyces octosporus* NCYC 427	dihydrocarvone dihydrocarveol	[69]
	*Trichosporum cutaneum* CCT 1903	dihydrocarveol epoxydihydrocarveol 6-isopropenyl-3-methyl-2-oxo-oxepanone isopropenyl-6-oxoheptanoic acid	[70]
	*Acinetobacter* sp. NCIB 9871 *Acinetobacter* sp. TD63	6-isopropenyl-3-methyl-2-oxo-oxepanone	[57]
	*Spodoptera litura*	7-hydroxycarvone 10-hydroxycarvone 10-hydroxycarveol 8,9-dihydroxy-8,9-dihydrocarvone	[71]
